# The Effect of Tailored Web-Based Feedback and Optional Telephone Coaching on Health Improvements: A Randomized Intervention Among Employees in the Transport Service Industry

**DOI:** 10.2196/jmir.4005

**Published:** 2016-08-11

**Authors:** Madeleine Solenhill, Alessandra Grotta, Elena Pasquali, Linda Bakkman, Rino Bellocco, Ylva Trolle Lagerros

**Affiliations:** ^1^ Karolinska Institutet Department of Medicine Clinical Epidemiology Unit Stockholm Sweden; ^2^ Halmstad University School of Health and Welfare Halmstad Sweden; ^3^ Karolinska Institutet Department of Medical Epidemiology and Biostatistics Stockholm Sweden; ^4^ Karolinska Institutet Department of Clinical Science Division of Baxter Novum, Intervention and Technology Huddinge Sweden; ^5^ University of Milano-Bicocca Department of Statistics and Quantitative Methods Milan Italy; ^6^ Karolinska University Hospital Huddinge Department of Medicine, Clinic of Endocrinology, Metabolism and Diabetes Stockholm Sweden

**Keywords:** diet, exercise, Internet, intervention studies, lifestyle, motivation, occupational health, questionnaires, randomized

## Abstract

**Background:**

Lifestyle-related health problems are an important health concern in the transport service industry. Web- and telephone-based interventions could be suitable for this target group requiring tailored approaches.

**Objective:**

To evaluate the effect of tailored Web-based health feedback and optional telephone coaching to improve lifestyle factors (body mass index—BMI, dietary intake, physical activity, stress, sleep, tobacco and alcohol consumption, disease history, self-perceived health, and motivation to change health habits), in comparison to no health feedback or telephone coaching.

**Methods:**

Overall, 3,876 employees in the Swedish transport services were emailed a Web-based questionnaire. They were randomized into: control group (group A, 498 of 1238 answered, 40.23%), or intervention Web (group B, 482 of 1305 answered, 36.93%), or intervention Web + telephone (group C, 493 of 1333 answered, 36.98%). All groups received an identical questionnaire, only the interventions differed. Group B received tailored Web-based health feedback, and group C received tailored Web-based health feedback + optional telephone coaching if the participants’ reported health habits did not meet the national guidelines, or if they expressed motivation to change health habits. The Web-based feedback was fully automated. Telephone coaching was performed by trained health counselors. Nine months later, all participants received a follow-up questionnaire and intervention Web + telephone. Descriptive statistics, the chi-square test, analysis of variance, and generalized estimating equation (GEE) models were used.

**Results:**

Overall, 981 of 1473 (66.60%) employees participated at baseline (men: 66.7%, mean age: 44 years, mean BMI: 26.4 kg/m^2^) and follow-up. No significant differences were found in reported health habits between the 3 groups over time. However, significant changes were found in motivation to change. The intervention groups reported higher motivation to improve dietary habits (144 of 301 participants, 47.8%, and 165 of 324 participants, 50.9%, for groups B and C, respectively) and physical activity habits (181 of 301 participants, 60.1%, and 207 of 324 participants, 63.9%, for B and C, respectively) compared with the control group A (122 of 356 participants, 34.3%, for diet and 177 of 356 participants, 49.7%, for physical activity). At follow-up, the intervention groups had significantly decreased motivation (group B: *P*<.001 for change in diet; *P*<.001 for change in physical activity; group C: *P*=.007 for change in diet; *P*<.001 for change in physical activity), whereas the control group reported significantly increased motivation to change diet and physical activity (*P*<.001 for change in diet; *P*<.001 for change in physical activity).

**Conclusion:**

Tailored Web-based health feedback and the offering of optional telephone coaching did not have a positive health effect on employees in the transport services. However, our findings suggest an increased short-term motivation to change health behaviors related to diet and physical activity among those receiving tailored Web-based health feedback.

## Introduction

Behaviors related to diet, physical activity, sleep, stress, and use of tobacco and alcohol play an important role in the prevention of lifestyle-related health problems such as obesity, diabetes, and cardiovascular diseases [1]. According to a review by *Lancet*, lifestyle interventions with a holistic perspective on health, such as an individual’s overall lifestyle behaviors, are more successful than those only focusing on one specific behavior [2]. Employees in the transport service industry represent one of many target groups that would benefit from tailored interventions focusing on healthy lifestyle behaviors. A needs assessment conducted by our research team among a sample from the Swedish transport service industry indicated that more than 60% of the track technicians were overweight, 70% had an unhealthy diet, and almost 50% had high cholesterol levels. Furthermore, a report by Kecklund et al [3] highlights health risks associated with irregular workdays due to shift work, long working hours, and the lack of recovery—common characteristics of Swedish transport employees.

We had previously reported findings on employees in the Swedish transport service industry. In detail, different methods to encourage study participation of this target group in a large Web-based lifestyle intervention study (described herein) were examined. The effects of email reminders and additional, more practical, reminders on overall study participation were specifically studied. Sending email reminders was an effective approach to encourage study participation in our intervention study [4]. Until now, we have not yet reported the health effects from our Web-based lifestyle intervention study.

To intervene at the workplace can be an effective health promotion strategy, which has also been endorsed by the World Health Organization which stated, “the workplace directly influences the physical, mental, economic and social well-being of workers and in turn the health of their families, communities and society. It offers an ideal setting and infrastructure to support the promotion of health of a large audience” [5]. However, it may be challenging to intervene at the workplace in the transport service industry because of different types of professions among the employees, resulting in different work tasks, schedules, and work settings. Tailored lifestyle interventions would therefore be ideal.

The advancements in technology allow for tailored lifestyle interventions. Prior research suggests promising results for Web-based physical activity interventions on improved physical activity habits [6-9]. The effect is even more valuable as Web-based interventions bridge the gap between the individual’s need for health interventions and the primary care’s lack of capability to support these individuals [9]. Mateo et al and Muntaner et al further support technologies to increase physical activity by using mobile phones, apps, and digital assistants [6,7]. Similar effects and types of interventions have been found for diet [10-12] and weight loss [13].

For instance, we had previously reported findings from a public Web-based weight loss club targeting behavioral change, using interactive progress charts, graphs, recipes, and chat forums [13]. We found that men and foremost participants older than 45 years were particularly successful in changing their eating behaviors, which was positively associated with greater weight loss [12]. In addition, our male participants logged in to the website most frequently, compared with our female participants. Related research carried out by our research team [14] builds on these findings, suggesting that an interactive food plate would serve as an appropriate tool to assess food intake among a male cohort via the Internet.

According to a review conducted by Webb et al [15], Web-based health interventions that are based on health behavior theories are more successful in promoting health behavior change, compared with nontheory-based health interventions. Also, Web-based interventions that incorporate more behavior change techniques and modes of delivery in addition to the Web further enhance the effects of a health intervention as opposed to fewer health behaviors techniques and 1 mode of delivery [15]. Yet, the actual effect of an intervention may also be related to the type of outcome variable measured [16].

A recent review [17] compared the effects from interventions using Web-based, printed materials, or the telephone to support healthier behaviors with respect to physical activity, diet, and weight control. Telephone intervention was particularly effective to promote healthier habits. Further research supports the telephone as an intervention tool in smoking and alcohol cessation programs [18-20]. In Sweden, there are mainly 2 helplines to encourage healthier behaviors among the public with respect to tobacco and alcohol. These are free of charge and have shown promising effects in reducing alcohol use and alcohol problems [19,21].

Consequently, the aim of this study was to evaluate the effect of tailored Web-based health feedback and the offering of optional telephone coaching with respect to improved health, in comparison to no health feedback or telephone coaching among employees in the transport service industry.

## Methods

### Participants

We asked 3,876 employees (18-65 years) at 4 transportation companies in the Swedish transport service industry to participate in a lifestyle health intervention. The invited companies represented (1) The Swedish Transport Administration (n=453), (2) The Swedish national train operator (n=2,391), (3) The local train operator in Stockholm (n=573), and (4) The subway transportation operator in Stockholm (n=459). Because the intervention study had a Web-based approach, only those employees with a work email were asked to participate.

### Study Design

The employees were emailed a link to the lifestyle health intervention with personal login details (username and password), information about the study, and instructions on how to participate in the study.

When the link was emailed, the participants were randomized to 1 of the 3 groups: (A) control group or (B) intervention Web or (C) intervention Web + telephone. All the 3 groups received an identical questionnaire on health and lifestyle behaviors, only the interventions differed. The intervention group B received tailored Web-based health feedback, and intervention group C received tailored Web-based health feedback + additional optional telephone health coaching for those participants who were motivated to change health behaviors. The Web-based feedback and the optional telephone coaching were based on the participants’ responses to the questions in the questionnaire. The CONSORT-EHEALTH has been used to report this study (Multimedia Appendix 1).

### Questionnaire

When the participants opened the link, they entered a welcome page with participant information and an informed consent form. By giving informed consent, they entered the questionnaire. Both the baseline and the follow-up questionnaires assessed the following 9 health areas divided into sections: body mass index (BMI), dietary intake, physical activity, stress, sleep, tobacco consumption, alcohol consumption, history of disease, and self-perceived health.

The questionnaire was originally developed to be filled out by using a computer, but it was possible to access the questionnaire using mobile phones.

### Outcome Measures

In details, our primary outcome was BMI (kg/m^2^), computed by dividing the body weight (in kilograms) by the height (in meters) squared. Secondary outcomes were: eating breakfast (times/week), carbohydrates intake (times/week), sugar intake (times/week), saturated fats intake (times/week), unsaturated fats intake (times/week), leisure-time physical activity (days/week), minutes of leisure-time activity/week (“0-10,” “11-20,” “21-30”, “31-40,” “41-50,” “51-60,” “>60” minutes/week), and total physical activity/day in metabolic equivalent of task (MET) hours, feeling stressed (“no,” “little,” “somewhat,” “quite a lot,” “very much”), sleeping ≤5 hours/night (“never,” “seldom,” “sometimes,” “frequently,” “mostly”), sleeping ≥9 hours/night (“never,” “seldom,” “sometimes,” “frequently,” “mostly”), feeling well-rested after sleeping, (“never,” “seldom,” “sometimes,” “often,” “mostly,” “always,” “do not know”), number of occasions of alcohol consumption (“never,” “1 time/month or less,” “2-3 times/month,” “2-3 times/week,” “≥4-6 times/week”), number of glasses during a typical alcohol occasion (“1-2 glasses,” “3-4 glasses,” “ ≥ 5 glasses”), smoke (“daily,” “sometimes,” “no”), number of cigarettes per week, use of moist oral snuff, “snus” (“daily,” “sometimes,” “no”), and number of packages of snus per week.

All questionnaire sections (besides self-perceived health and BMI) ended with a follow-up question on the participants’ “motivation to change” their health habits in that specific health area. The response options for these question was in accordance with Prochaskas’ transtheoretical model (TTM) of behavior change using the 5 phases—precontemplation, contemplation, preparation, action, and maintenance—to assess participants’ readiness to change now, within a month, within 6 months, not now, or not at all [22]. If the participants were motivated to change their health habits, they were recommended to contact any of the telephone helplines to receive coaching for healthier habits, presumed that they were assigned to group C intervention Web + telephone (see more details in the following section).

### Development of Questionnaire and Randomization

The researchers developed the questionnaire and the content of the feedback and were involved in the development of content of the telephone coaching. We asked experts in respective health field to approve the questions asked, and its feedback, for each part of the questionnaire. The questionnaire was pilot tested by truck drivers, the target group, and the research unit at Karolinska Institutet. Most questionnaire sections used validated items from previous research. We used a questionnaire to assess food frequency including the intake of carbohydrates or fibers; sugar; saturated fats or high fat intake; and unsaturated fats or low fat intake. To complement the food frequency questionnaire, we used an interactive virtual food plate developed by the research team to assess lunch intake, by allowing the participants to complete a typical lunch meal by dragging pictures of food items to a virtual food plate. This interactive food plate has been validated and described previously [14]. Alcohol consumption was studied using the Alcohol Use Disorders Identification Test, AUDIT-C [23], and tobacco use was assessed using validated questionnaires provided by the Swedish National Tobacco Quitline. Sleep was assessed using the validated Karolinska Sleep Questionnaire [23]. Physical activity was measured using a validated questionnaire for total energy expenditure [24]. The instrument has 9 ordered intensity levels, each assigned a value expressed as a multiple of metabolic energy turnover [25] and exemplified by common activities. The participants reported the time spent on each intensity level, in total 24 hours, which allowed for an estimate of MET hours per day. In addition, we asked the participants to indicate the average number of times per week spent on leisure-time physical activities such as brisk walking, running, aerobics or weight lifting and skiing, and number of minutes per session. These self-reported questions about leisure-time physical activity are often used in epidemiological research, for example, the question about leisure-time activity is similar to the questions used in studies by McTiernan et al [26] and Godin et al [27] and extensively validated against other measures such as maximal oxygen uptake and accelerometers.

A Web company [28] was responsible for developing a Web-based version of the research material in collaboration with the research group. They were also responsible for administrating the website enclosing the feedback to ensure overall feasibility and usability. A helpdesk was administered by the research group and the Web company if participants needed any help to access or use the website or had any questions regarding the content of the feedback.

The research group collected lists of potential participants (employees) from the company manager at the 4 companies. All employees with an email address were eligible for the study. The participant lists were given to the Web company, which was responsible for distributing the questionnaires. Randomization lists were automatically created by the Web company before distributing the questionnaire link.

Reminders to take part in the study were sent to all nonresponders to encourage study participation at baseline and follow-up, which has been described in detail previously [4]. In brief, a total of 4 to 5 and 11 email reminders were sent at baseline and follow-up, respectively. The difference of reminders (4 or 5) depended on when the participants entered the study. The email reminders increased the total response rate by 15% and 21% at baseline and follow-up, respectively. Additional reminders such as informational texts about the study, fliers, oral presentations, and visits by the research group as well as texting were also distributed in addition to the email reminders. Thus, we had several strategies to encourage our target group to participate in this intervention study.

Only those who submitted the questionnaire(s) were considered participants in this study.

### Intervention Web

**Figure 1:**
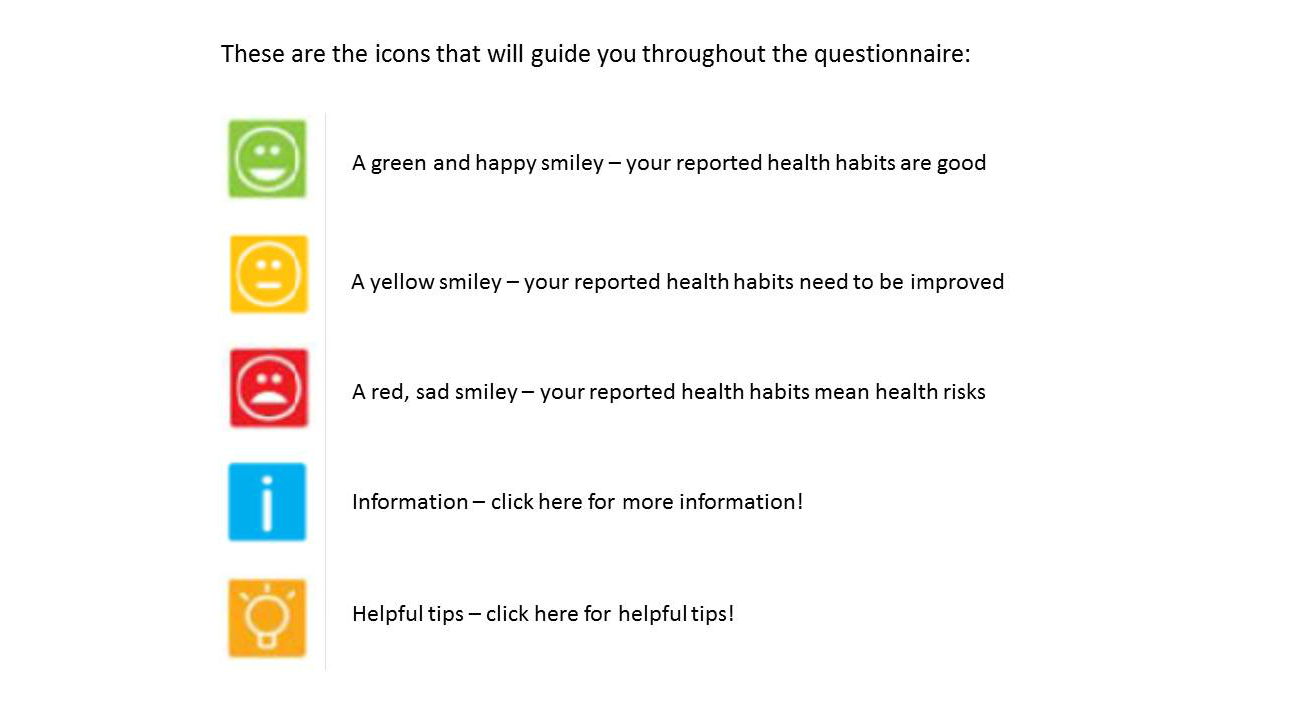
Screenshot of the icons used to guide the participants throughout the completion process of the questionnaire.

The participants received icons such as colored smiley faces and icons symbolizing “information” and “advice” to guide them throughout the completion process of the questionnaire and with respect to health changes. The smiley faces provided the participants with tailored feedback on level of satisfaction regarding the reported health habit according to national health guidelines [29]. A green and happy smiley indicated “your reported health habits are good”; a yellow smiley indicated “your reported health habits need to be improved”; a red, sad smiley indicated “your reported health habits mean health risks.” The icons for “information” and “advice” allowed the participant to click forward to get more information about the health habit and to receive specific advice and recommendations to improve that specific health habit (see Figure 1).

**Figure 2:**
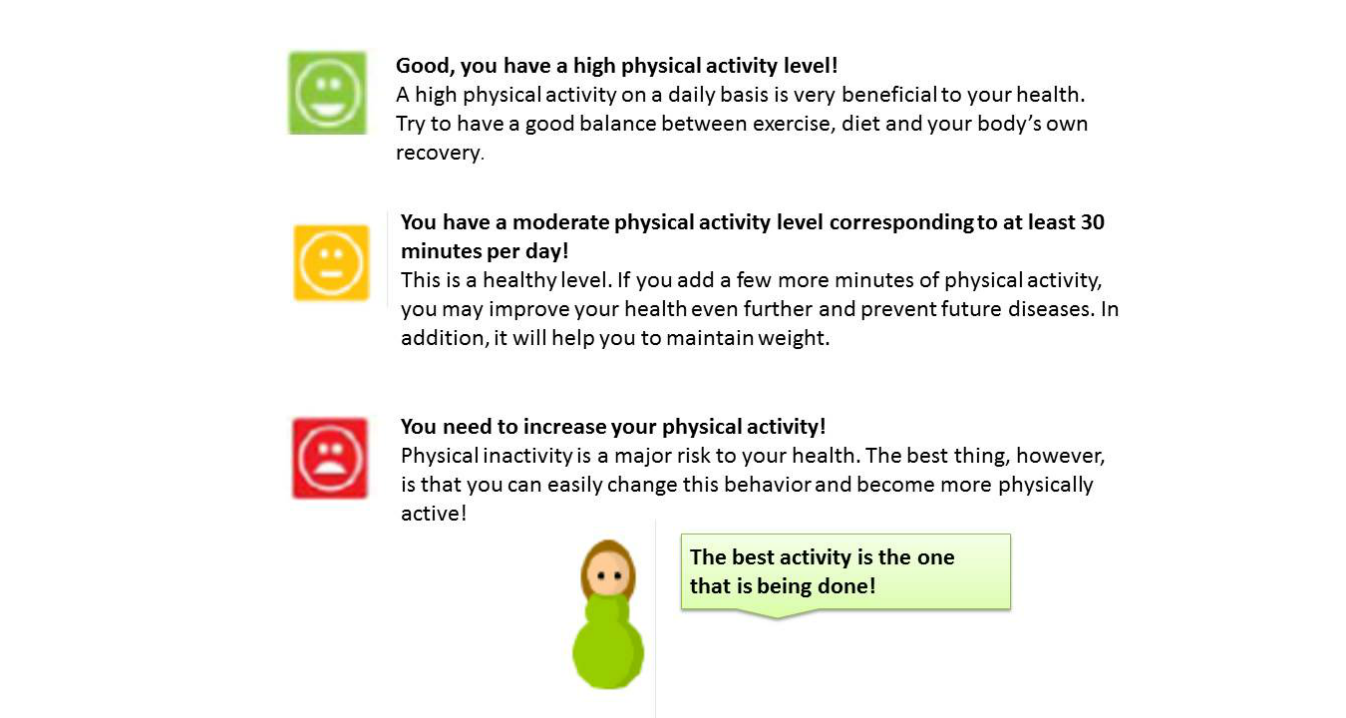
Screenshot of examples of the tailored web-based health feedback given for physical activity.

In addition to the icons shown in Figure 1, tailored feedback appeared instantly and automatically on the computer screen after the participants had responded to each of the sections with questions (Figure 2). For example, if the participants reported a low physical activity level, that is, not meeting the national health guidelines [29], information about the significance of being physically active and advice on how to increase physical activity as a natural part of life were provided via the computer screen. The participants were able to read the feedback instantly. All feedback was summarized and saved at the end of the questionnaire and on a tailored website, which the participants could visit at any time using their personal login details.

### Intervention Web + Telephone

**Figure 3:**
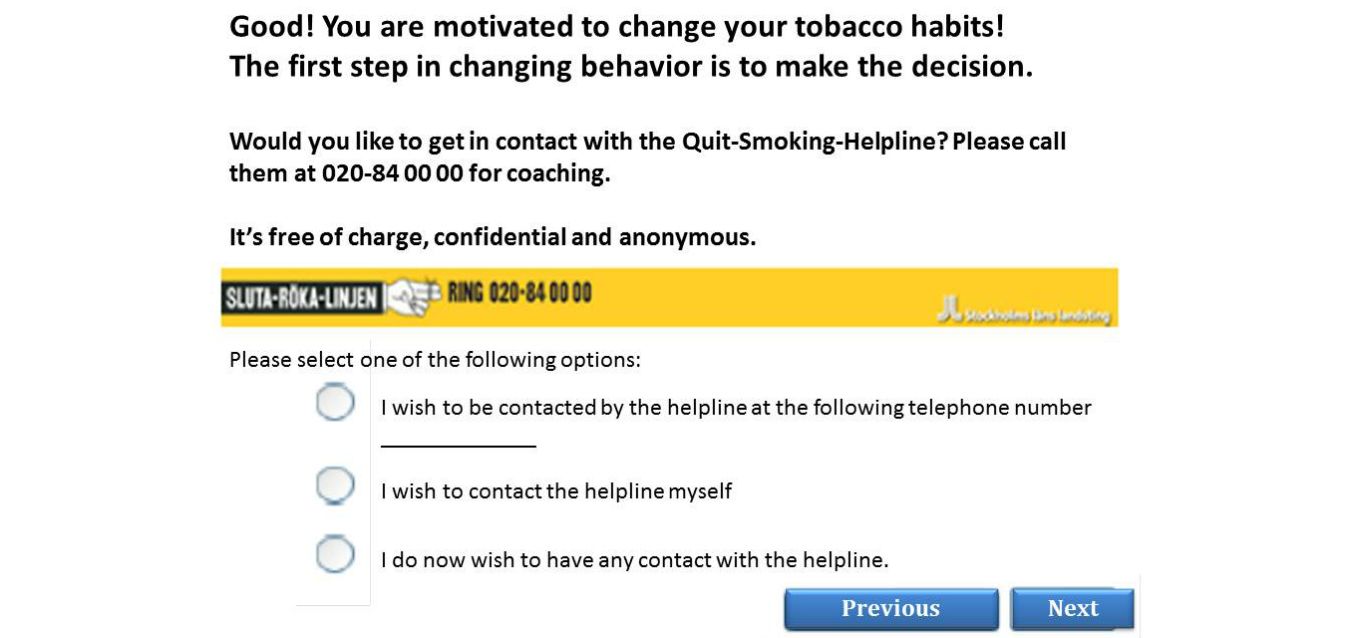
Screenshot of referral to The Swedish National Tobacco Quitline.

The participants were offered the opportunity for telephone coaching in addition to Web-based tailored health feedback, if their reported health habits did not meet the national guidelines or if they expressed motivation to change health habits. If the participants were referred to telephone coaching, they could either leave their mobile phone number to be dialed (reactive help) by the telephone helplines or dial themselves (proactive help) (Figure 3). The 3 helplines that were offered were: (1) The Swedish National Tobacco Quitline, (2) The Swedish National Alcohol Helpline, and (3) The Diet and Exercise Helpline. The helplines for tobacco and alcohol are well-established public helplines in Sweden [30]. The Diet and Exercise Helpline was developed for this study. All helplines used counselors who were specifically trained to work effectively with behavior changes using traditional counseling approaches such as the motivational interviewing technique [31] and TTM [22].

The tailored Web-based health feedback and the telephone coaching services were available during 9 months. After 9 months, all participants received a follow-up questionnaire including intervention Web + telephone coaching. The following differences between baseline and follow-up applied: at follow-up, the participants had access to the website encompassing their personalized feedback. This website was however only available during 2 weeks after the study had ended. All participants were offered telephone coaching by The Swedish National Tobacco Quitline and The Swedish National Alcohol Helpline but not by the Diet-and Exercise Helpline. The reasons for this were that the helplines for tobacco and alcohol are national public health services, whereas the helpline for diet and exercise habits was part of the intervention itself and led by the research group.

Data were collected by the Web company [28] and sent to the researchers after the intervention had ended.

**Figure 4:**
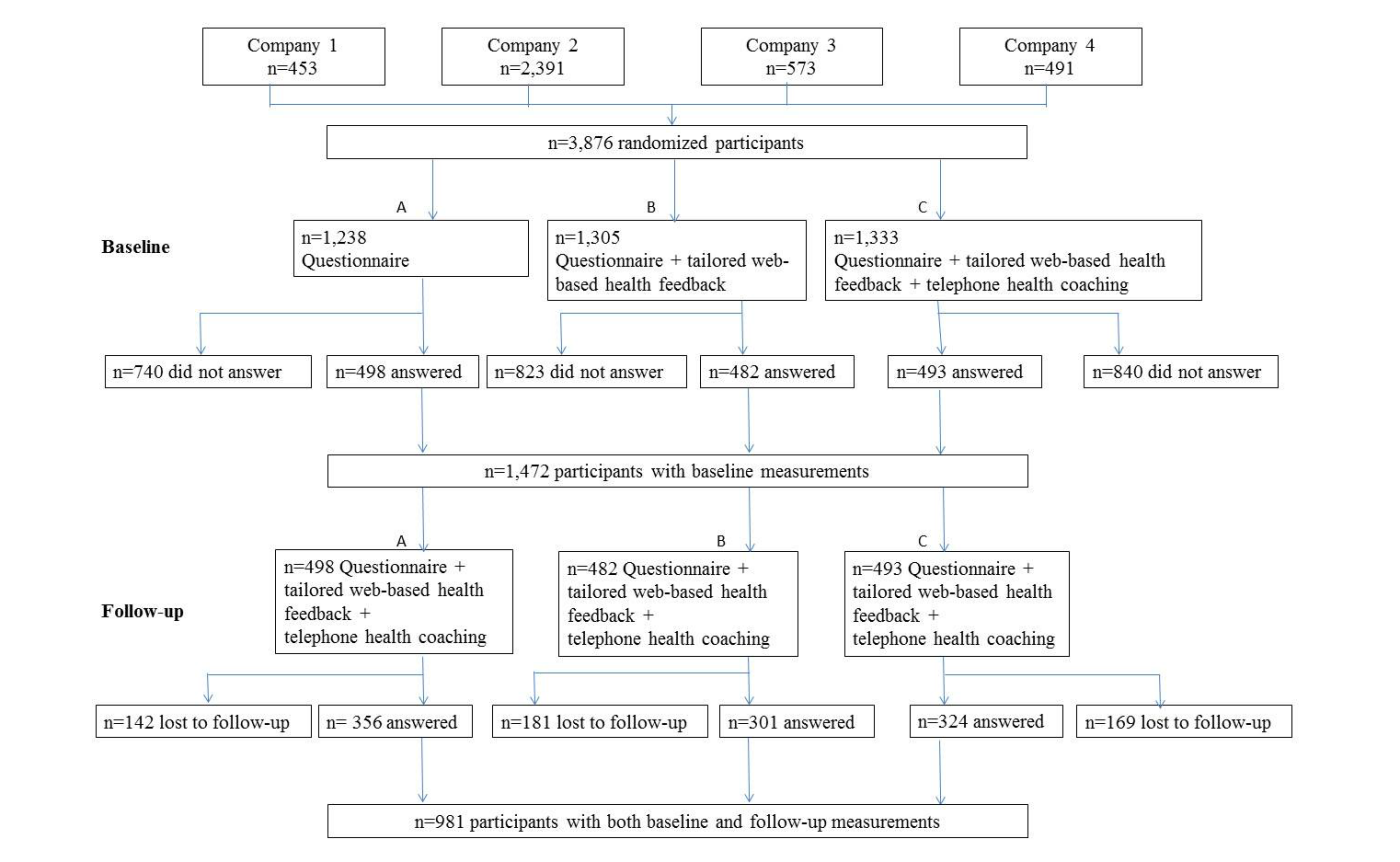
Flowchart of the study design. A total of 3876 employees were asked to participate in the study and were automatically randomized to 1 of the 3 groups: (1) control group or (2) intervention Web, or (3) intervention Web + telephone. All the 3 groups received an identical questionnaire on health and lifestyle behaviors, only the interventions differed. Nine months later, all participants received a follow-up questionnaire and interventions web + telephone.

The Ethics Committee of the Karolinska Institutet, Stockholm, Sweden, approved this study. See Figure 4 for a flowchart of the study design.

### Statistics

Descriptive statistics (proportions, medians, and means with standard deviations) of the participants’ characteristics (sex, age, BMI, physical activity level, use of tobacco—snus and smoking—and perceived health) at baseline (all sample, completers, noncompleters) and follow-up were computed. We also categorized the participants into office workers or field workers (if mostly office based=office worker; if mostly non–office-based with limited access to a computer=field worker). The participants’ referral and usage of the 3 telephone helplines were also examined.

We studied whether differences in participants’ reported health habits at baseline and follow-up varied across the 3 groups (A, B, and C). Chi-square tests were performed to study possible differences in categorical variables and analysis of variance (or Kruskal–Wallis) for continuous variables.

The participants’ levels of “motivation to change” were specifically studied. Both at baseline and follow-up, the participants answered the question “are you motivated to change your X behaviors,” in which X refers to the specific health behavior that was examined (ie, physical activity, diet, tobacco, or alcohol). The response options to these questions were: “yes, within a month,” “yes, within six months,” “yes, but in the future,” “do not know,” and “no.” We summarized the proportions of responses within the yes-options and the proportions within “do not know” and “no,” separately.

We used generalized estimating equation (GEE) to study specific effects of the interventions over time in relation to changes in reported health habits and motivation to change. GEE models are an extension of the simple regression model to contexts in which the outcome can be noncontinuous and measured more than once on the same subject. The GEE models were adjusted for age and gender.

Only those who participated at both baseline and follow-up were considered for analyses. STATA version 13.1 (Statacorp LP, College Station, TX) for Windows was used for all statistical calculations and analyses. All reported *P* values were 2-sided. *P*<.05 was considered statistically significant.

## Results

### Basic characteristics of the study participants

In total, 1473 of 3876 (38.00%) employees completed the Web-based lifestyle questionnaire, and 981 of 1473 (66.60%) employees participated both at baseline and follow-up. Table 1 summarizes baseline characteristics of subjects who started the study, stratified by being, or not being, lost to follow-up. At baseline, 984 of 1473 (66.80%) participants were men. They had a mean age of 44 years (SD 10.2), a BMI of 26.4 kg/m^2^(SD 4.2), and perceived their personal health as “rather good” or better. In total, 500 of 1473 (33.94%) participants were categorized as office workers, the remaining as field workers. There were no substantial differences between completers and noncompleters, even if subjects who were lost to follow-up were slightly younger and more inclined to smoke and use snus than subjects who proceeded in the study.

**Table 1:** The participants’ basic characteristics at baseline (all sample, completers, and noncompleters) and follow-up.

Characteristic	Baseline (all), N=1473^a^	Baseline (completers), N=981^b^	Baseline (non-completers), N=492^c^	Follow-up, N=981^b^
Sex, male (%)	984 (66.80)	655 (66.7)	329 (66.9)	656 (66.8)
Age, years (SD^f^)	43 (10.68)	44 (10.2)	42 (11.5)	44 (10.2)
Physical activity level/day, MET^e^hours (SD)	44.1 (13.66)	43. 8 (12.8)	44.8 (15.2)	44.0 (12.1)
Smoking, yes (%)	261 (17.72)	158 (16.1)	103 (20.9)	154 (15.7)
Snus, yes (%)	255 (17.31)	160 (16.3)	95 (19.3)	167 (17.0)
BMI^d^, kg/m^2^(SD)	26.3 (4.25)	26.4 (4.3)	26.1 (4.1)	26.5 (4.2)
Perceived health habits as “rather good” or better, yes (%)	1149 (78.00)	769 (78.4)	380 (77.2)	803 (81.8)

^a^Number of subjects who participated at baseline.

^b^Number of subjects who participated at both baseline and follow-up.

^c^Number of subjects who participated at baseline but were lost to follow-up.

^d^BMI: body mass index.

^e^MET: metabolic equivalent of task.

^f^SD: standard deviation.

### Interventions

Among the 981 employees who participated at both baseline and follow-up, 357 of 981 (36.4%) represented group A (control group), 301 of 981 (30.7%) group B (intervention Web), and 324 of 981 (33.0%) group C (intervention Web + telephone). See Figure 4.

In group C, at baseline, 173 (53.4%), 23 (7.1%), and 19 (5.8%) of 324 participants reported a wish to be dialed by or to dial themselves to the Diet and Exercise Helpline, the Swedish National Tobacco Quitline, and the Swedish National Alcohol Helpline, respectively. In details, 77 of 173 (44.5%) participants asked to be dialed by the Diet and Exercise Helpline, 9 of 23 (39.1%) participants by The National Tobacco Quitline, and 5 of 19 (26.3%) participants by The Swedish National Alcohol Helpline. All participants were contacted accordingly. The remaining participants reported that they would contact the helplines themselves. No participant contacted the Diet and Exercise Helpline voluntarily.

At baseline, 550 of 981 participants (56.1%) reported “do not know” or “no” motivation to improve dietary habits. Intervention groups B and C reported higher motivation to improve dietary habits (144 of 301 participants, 47.8%, and 165 of 324 participants, 50.9%, for groups B and C, respectively) and physical activity habits (181 of 301 participants, 60.1%, and 207 of 324 participants, 63.9%, for groups B and C, respectively) compared with the control group A receiving no health feedback (122 of 356 participants, 34.3%, for diet and 177 of 356 participants, 49.7%, for physical activity). At follow-up, the intervention groups had significantly decreased motivation (group B: *P*<.001 for change in diet; *P*<.001 for change in physical activity; and group C: *P=*.007 for change in diet; *P*<.001 for change in physical activity), whereas control group A reported a significant increase in motivation to change over time, at time of receiving health feedback in the follow-up questionnaire (*P*<.001 for change in diet; *P*<.001 for change in physical activity). See Table 2.

**Table 2:** Motivation to change diet and physical activity at baseline and follow-up across groups.

Motivation		Group A (n=356), Control		Group B (n=301), Intervention Web		Group C (n=324), Intervention Web + telephone	
		Baseline	Follow-up	Baseline	Follow-up	Baseline	Follow-up
**Plan to change diet, n (%)**							
	Yes, within 1 month	58 (16.3)	85 (23.9)	78 (25.9)	66 (21.9)	94 (29.0)	65 (20.1)
	Yes, within 6 months	31 (8.7)	32 (8.9)	31 (10.2)	32 (10.6)	38 (11.7)	40 (12.3)
	Yes, but not now	33 (9.2)	38 (10.7)	35 (11.6)	25 (8.3)	33 (10.2)	34 (10.5)
	Do not know	119 (33.4)	100 (28.1)	95 (31.6)	98 (32.5)	85 (26.2)	99 (30.5)
	No	115 (32.3)	101 (28.3)	62 (20.6)	80 (26.6)	74 (22.8)	86 (26.5)
*P* for change^a^		<.001		<.001		.007	
**Plan to change physical activity, n (%)**											
	Yes, within 1 month	96 (26.9)	113 (31.7)	106 (35.2)	91 (30.2)	114 (35.2)	91 (28.1)
	Yes, within 6 months	38 (10.7)	36 (10.1)	41 (13.6)	31 (10.2)	52 (16.1)	43 (13.3)
	Yes, but not now	43 (12.1)	40 (11.2)	34 (11.3)	43 (14.3)	41 (12.6)	36 (11.1)
	Do not know	79 (22.2)	61 (17.1)	53 (17.6)	65 (21.6)	39 (12.0)	56 (17.3)
	No	100 (28.1)	106 (29.8)	67 (22.2)	71 (23.6)	78 (24.1)	98 (30.2)
*P* for change^a^		<.001		<.001		<.001	

^a^p-value obtained from GEE models.

Participants’ health habits at baseline and follow-up are reported in Multimedia Appendix 2. When analyzing possible changes between baseline and follow-up measurers, no significant differences between groups were found. Our results from the GEE models support no significant changes over time for the various reported health aspects at baseline and follow-up or between the 3 groups. However, our results suggest significant increases in days of eating breakfast (*P*<.001), days of physical activity per week (*P*=.002), and decreases in sugar intake (*P*<.001) at follow-up, with no statistically significant differences regarding type of interventions (group B or C).

## Discussion

The results from this randomized Web-based intervention suggest no significant health improvements from tailored Web-based health feedback and the offering of optional telephone coaching, in comparison to no health coaching among employees in the Swedish transport industry.

However, our findings point toward an impact of automatic tailored health feedback on increased motivation to change behaviors. More specifically, at baseline, the intervention groups receiving feedback reported higher motivation to improve dietary and physical activity habits compared with the control group A. At follow-up, all the groups received feedback, but only the control group, now receiving feedback, reported an increased motivation to change dietary and physical activity habits. It may thus be proposed that tailored health feedback positively alters participants’ motivation to engage in healthier lifestyle habits, but, the effect does not last over time, not even if the feedback is offered again.

The results from our study are in line with the findings from a randomized Web-based intervention study examining participants’ motivation to improve cardiovascular health conducted by Ayres et al [32]. In short, they randomized their participants to receive either motivational feedback in the form of a personalized risk assessment of their current cardiovascular health or to respond to questions assessing intentions, attitudes, and anticipated regret about managing cholesterol levels through diet. They also randomized their participants to a group combining these 2 interventions or to a control group receiving no intervention. Their results indicate an enhanced motivation to improve cardiovascular health from the combined intervention (personalized health feedback and questions on intentions to change health behavior). The results of our study may therefore be supported by this study; in this study, an amplified effect of tailored feedback was found, even if it was for a short term [32]. However, it is difficult to draw conclusions on the actual cause and effect relationship. Prior research suggests a question–behavior effect, meaning that simply asking a person to answer questions about the intention to change behavior may positively influence a person’s process to change that behavior [33]. For instance, participants may then reflect on the registered health aspects, which in turn may lead to an increased motivation to change them. Because many Web-based intervention studies use both interventions and questions as part of their design, it is difficult to study whether it is the act of responding to questions or the intervention itself—or a combination of both—that may cause the effect [34].

Prior Web-based lifestyle interventions using tailored health feedback report modest effects on health improvements [35], and previously published systematic reviews of non–Web-based health promotions conducted among workplace personnel show inconclusive results [36]. Also, few Web-based lifestyle interventions have been conducted among a target group such as ours with predominantly middle-aged overweight men in the transport industry, making it difficult to find support from previous research. Pressler et al report no increased effects from Web-based interactive feedback to promote physical activity among sedentary employees at an automobile company [37], compared with ordinary personal advice to encourage physical activity. Hansen et al, however, found an improved level of leisure-time physical activity from their Web-based population-based intervention study after 3 and 6 months but report no other health improvements such as reductions in BMI, waist circumference, body fat percentage, or blood pressure [8].

Schulz et al indicate small health behavior improvements for physical activity, vegetable and fruit consumption, alcohol intake, and tobacco consumption. They specifically studied differences in health effects from encouraging their participants with either sequential health behavior changes (one at the time) or simultaneous health behavior changes (all at the same time), suggesting that sequential health changes produce greater effects the first year, but that simultaneous changes generate a more long-term effect found after 2 years [38]. These findings are in line with those of research by Krebs et al, suggesting that tailored interventions using iterative feedback enhance the effects when compared with tailored interventions based on “one assessment feedback only” [35]. In our study, we asked the participants to change all health behaviors at once, meaning that the potential long-term effects on health behavior improvements might be too early to detect from our 9-month intervention. Research studies indicate higher dropout rates for interventions promoting simultaneous behavior changes due to prolonged time for the participants to see individual health improvements [39], which could support the response rate in our study [4].

Our participants who received Web-based health feedback received it instantly when filling out the questionnaire and, if they wanted to, had the opportunity to read it again on the website after submitting their questionnaire. It is possible that once they had read the feedback on the screen (and potentially saved it or printed it), there might not have been a reason to revisit the website because it offered no new tips, updated information, or interactive features such as chat rooms or blogs. The fact that 66% of the study participants were field workers may have influenced the actual effect on the intervention. Even if all study participants had access to computers and mobile phones during their workday, it is possible that field workers were less likely to enter the website enclosing the tailored feedback, due to inconveniences of their work setting from being out in the field. It would be interesting to study how participants would use a questionnaire such as ours with interactive feedback as a continuous mean to assess their own health and receive instant feedback on new health behaviors. However, it is complicated to draw conclusions from interactive data collection, including our results.

Even if the questionnaire was exactly the same in the 3 groups, we cannot rule out that the intervention groups were affected by the fact that they received instant feedback. Thus, although randomization was conducted to produce comparability between the groups with respect to unmeasured confounding, the feedback in itself may have introduced a difference. Results such as comparisons at baseline and completers’ analyses should therefore be interpreted with caution.

Moreover, the follow-through on the “opt in” and “I will call you” feature for contact with the telephone helplines in this study is interesting and novel. It appears that 55% to 74% of participants interested in telephone coaching indicated that they would contact the helplines on their own initiative; however, no one voluntarily contacted the helplines. Perhaps it requires a stronger courage by the individual to dial a helpline to ask for help, as opposed to putting the responsibility in the hands of the health counselors. Future research is recommended to study this further, particularly as nonfacial health interventions are rapidly increasing in popularity due to the advancement of technology. Many interventions are indeed based on the individuals’ own capacity to take the first step in his or her behavior change.

The contact information to the 3 helplines was only provided in the questionnaire and thus saved at the personalized website. The helplines for tobacco and alcohol were, on the other hand, also advertised at general health care settings as they were open to the public, but the information to the Diet and Exercise helpline was only provided in the questionnaire. Frequent reminders and information about the feedback and telephone helplines could have increased the use of these features and the number of callers to the 3 helplines.

Even if our telephone coaching used well-established coaching techniques such as the motivational interviewing technique, the benefits of the technique may not have been fully realized because telephone coaching was only offered to those with “higher” levels of motivation to change health behaviors. In fact, the strength of motivational interviewing is the ability to explore ambiguity and to help individuals develop stronger motivation and an interest in behavior change. Thus, our study design might have restricted us to fully make use of this coaching technique, which in turn may have influenced the actual intervention effect of telephone coaching.

We did not track the participants’ logins to the tailored website enclosing the personal health feedback, which would have allowed us to study the participants’ use of the website. We have previously reported findings on the association between number of logins to a Web-based weight club and greater weight loss among men [13]. Furthermore, van Genugthen et al suggest that participants with adequate skills to self-regulate their health behaviors and who possess action planning skills are more likely to visit a tailored Web-based intervention compared with participants with less self-regulation and action planning skills [40]. However, Robroek et al state that even if web-based messages may increase website use, the actual use of the website’s tools might be low [41]. Thus, future studies are strongly recommended to invest time in promoting and documenting the participants’ use of interventions. Careful tracking may not only improve the quality of the study but also ensure participants’ partaking of proposed interventions.

We have previously presented results on response rate in this study, suggesting that the overall response rate may be vulnerable to specific occupational groups with less screen time, season of study, and the possibility to participate during work hours [4]. The method of using the Internet to deliver health interventions seems however appropriate for our study population. All participants in our study had email addresses and access to computers or mobile phones during time of study. About 90% of the Swedish population has access to the Internet, and 85% has broadband at home. A high computer literacy and experience of use among our study population is therefore likely.

There is no reason to believe that selection bias of highly motivated participants affected this study. Most of the participants were not motivated to change their dietary and physical activity habits at baseline. However, all the measures in this study rely on self-reports, which is a limitation in this study. Future studies would benefit from adding device-based measures, for example, for assessing physical activity. For instance, accelerometers can be used for large surveys at acceptable costs and to provide rather reliable measures [42]. Yet, the advancement in device-based measurers has escalated the past years and at the time of planning this study, little research using these devices had been done, thus explaining the choice of method of this Web- and telephone-based intervention study.

It cannot go unnoticed that our study sample represents a challenging subgroup in health research. Our participants represented mostly middle-aged men working in the transport industry. Extending our findings to other populations is therefore difficult. According to prior literature, overweight, middle-aged men are generally underrepresented in epidemiological lifestyle studies, supporting our results on total response rate [43]. In addition, previous research indicates that men have a tendency to procrastinate until they seek health care [44]. Therefore, any health improvement on our study group, such as increased motivation found in this study is of significance. The optimal intervention still depends on the individual’s motivation, personal preference, and level of implementation [38]. Consequently, even if we did not find any health effects from using tailored Web-based health feedback and optional telephone coaching as intervention tools to promote healthier lifestyle behaviors in our target group, it may have increased our participants’ motivation to adopt healthier dietary and physical activity habits. Future research is recommended to further study the effects on motivation with respect to short-term and long-term effects.

### Conclusion

Tailored Web-based health feedback and the offering of optional telephone coaching did not have a positive health effect on employees in the transport service industry. However, our findings suggest an increased short-term motivation to change health behaviors related to diet and physical activity among those receiving tailored Web-based health feedback.

## Appendix

Multimedia Appendix 1 CONSORT eHealth checklist V 1.6.1.

Multimedia Appendix 2 The participants’ reported health at baseline and follow-up (n=981).

## Abbreviations

BMI: body mass index
GEE model: generalized estimating equation regression models
MET: metabolic equivalent of task
TTM: transtheoretical model
