# Supplementary material for: The Effect of Tailored Web-Based Feedback and Optional Telephone Coaching on Health Improvements: A Randomized Intervention Among Employees in the Transport Service Industry
Source: J Med Internet Res. 2016 Aug 11;18(8):e158. doi: 10.2196/jmir.4005 (PMC4999534; doi:10.2196/jmir.4005)
Supplement: Multimedia Appendix 2 [file jmir_v18i8e158_app2.pdf]

Supplemental file

Table 3. The participants' reported health at baseline and follow-up (n=981).

Health behaviors							Comparison of differences between follow-up and baseline among groups[1]
	Group A (n= 356) *Control*		Group B (n=301) *Intervention web*		Group C (n=324) *Intervention web + telephone*		p-value
	Baseline	*Follow-up*	*Baseline*	*Follow-up*	*Baseline*	*Follow-up*	
Body composition BMI, kg/m^2, mean (SD)[2]	26.6 (4.5)	26.6 (4.6)	26.6 (4.4)	26.5 (4.1)	26.1 (4.0)	26.2 (4.0)	0.36
Dietary intake[3] Eat breakfast, days/week, mean (SD)	6.2 (1.6)	6.47 (1.2)	6.2 (1.7)	6.5 (1.3)	6.3 (1.5)	6.49 (1.2)	0.26
Carbohydrates, times/week, mean (SD)	21.5 (8.2)	22.1 (8.3)	23.2 (8.0)	22.8 (8.2)	22.7 (8.0)	22.37 (8.6)	0.10
Sugar, times/week, mean (SD)	9.1 (5.5)	8.4 (5.7)	8.5 (5.3)	8.1 (5.1)	9.0 (5.3)	8.06 (5.0)	0.19
Saturated fats, times/week, mean (SD)	16.8 (7.4)	16.5 (7.1)	16.4 (7.2)	16.6 (6.7)	16.9 (6.9)	16.97 (6.5)	0.57
Unsaturated fats , times/week, mean (SD)	9.1 (5.0)	9.6 (5.1)	9.8 (5.1)	10.3 (5.4)	9.7 (5.2)	10.0 (5.4)	0.85
Physical activity days/week, mean (SD)	2.1 (1.9)	2.3 (1.9)	2.2 (1.8)	2.3 (1.8)	2.3 (1.9)	2.6 (2.0)	0.36
min/these days, mean (SD)	50.7 (17.0)	50.4 (17.0)	48.2 (17.2)	48.0 (17.7)	50.3 (18.0)	50.7 (17.3)	0.67
Stress Feel stressed now "quite a lot" or "very much" *n* (%)	42 (11.8)	35 (9.8)	33 (11.0)	22 (7.3)	29 (8.9)	26 (8.0)	0.82
Sleep ≤5 hours/night, "frequently" or "mostly" *n* (%)	58 (16.3)	49 (13.8)	57 (18.9)	55 (18.3)	55 (17.0)	41 (12.7)	0.36
≥9 hours/night, "frequently" or "mostly" *n* (%)	50 (14.0)	45 (12.6)	41 (13.6)	32 (10.6)	36 (11.1)	26 (8.0)	0.12
Feel well-rested after sleeping, "often", "mostly" or "always" *n* (%)	107 (30.1)	98 (27.5)	73 (24.2)	71 (23.6)	80 (24.7)	84 (25.9)	0.17
Alcohol intake No of occasions of alcohol consumption*, *n* (%)							0.40
Never	15 (4.2)	19 (5.3)	19 (6.3)	19 (6.3)	18 (5.6)	18 (5.6)	
1 time/m or less	97 (27.2)	91 (25.6)	82 (27.2)	79 (26.2)	76 (23.5)	90 (27.8)	
2-3 times/month	179 (50.3)	183 (51.4)	144 (47.8)	146 (48.5)	185 (57.1)	176 (54.3)	
2-3 times/week	60 (16.8)	56 (15.7)	53 (17.6)	52 (17.3)	38 (11.7)	32 (9.9)	
≥4-6 times/week	5 (1.4)	7 (2.0)	3 (1.0)	5 (1.7)	7 (2.2)	8 (2.5)	
Number of glasses during a typical alcohol occasion, *n* (%)							0.59
1-2 glasses	129 (38.0)	144 (43.0)	113 (40.5)	129 (46.1)	126 (42.0)	132 (43.8)	
3-4 glasses	121 (35.7)	120 (35.8)	116 (41.6)	112 (40.0)	113 (37.7)	114 (37.9)	
≥ 5 glasses	89 (26.2)	71 (21.2)	50 (17.9)	39 (13.9)	61 (20.3)	55 (18.3)	
Tobacco Current smoking, "sometimes" or "daily" *n* (%)	67 (18.8)	69 (19.4)	47 (15.6)	42 (14.0)	44 (13.6)	43 (13.3)	0.59
No of cigarettes per week, mean (SD)	14.4 (18.1)	23.6 (21.0)	17.2 (17.5)	15.5 (13.8)	19.4 (18.5)	23.6 (21.0)	0.56

[1] ANOVA (or Kruskal Wallis) for continuous variables; Chi-square test for categorical variables.
[2] Standard deviation.
[3] Questions assessing the intake of similar dietary components have been merged and computed to times/week.
